# Severe claw lesions in pregnant sows reduce prolificacy and increase litter heterogeneity

**DOI:** 10.1186/s40813-025-00462-5

**Published:** 2025-10-24

**Authors:** Henar Gonzalez-Ramiro, Adelina López-Jara, Josep M. Cambra, Alejandro Gonzalez-Plaza, Manuela Garcia-Canovas, Marina Lopez-Arjona, Maria Botia, Maria A. Gil, Cristina Cuello, Heriberto Rodriguez-Martinez, Emilio A. Martinez, Inmaculada Parrilla

**Affiliations:** 1 Department of Medicine and Animal Surgery, Faculty of Veterinary Medicine, International Excellence Campus for Higher Education and Research, University of Murcia - Institute for Biomedical Research of Murcia, Campus de Ciencias de la Salud, Murcia, Spain; 2 Department of Research and Development, Grupo Agropor I+D+I, AIE, Murcia, Spain; 3 Interdisciplinary Laboratory of Clinical Analysis (INTERLAB-UMU), Department of Medicine and Animal Surgery, Faculty of Veterinary Medicine, International Excellence Campus for Higher Education and Research, University of Murcia, Murcia, Spain; 4 Department of Biomedical & Clinical Sciences (BKV), BKH/Obstetrics & Gynecology, Faculty of Medicine and Health Sciences, Linköping University, Linköping, SE-58185 Sweden

**Keywords:** Claw lesion, Litter heterogeneity, Stress biomarkers, Sows

## Abstract

**Background:**

Claw lesions (CLs) are highly prevalent in sow herds, affecting animal welfare and productivity. This study aimed to evaluate the incidence of CLs in pregnant sows and their impact on litter performance. Additionally, we assessed hair cortisol, cortisone, and oxytocin levels to determine whether CLs cause any stress in sows. The study involved 693 hyper-prolific sows, initially assessed for CLs at farrowing. After weaning, they were housed in individual AI crates, where their hair was shaved. At 28 days post-insemination, pregnant sows were moved to group pens. One week before expected farrowing, sows (*n* = 507) were transferred to individual farrowing crates where newly grown hair samples were collected, and CLs reassessed. Claw lesion severity was scored from 0 to 3 (SS0: no lesions, SS1: mild, SS2: moderate, SS3: severe). Sows were then classified into 3 categories (CAT): CAT1 (SS0 + SS1), CAT (SS1 + SS2, not SS3), and CAT3 (SS1 + SS2 + SS3). The total number of CL per sow and the final sow score were calculated as the sum of CLs observed on the claws and the sum of all CL severity scores, respectively.

**Results:**

Only 4,1% of sows had no CLs, while 66.5% exhibited moderate to severe lesions (CAT2+CAT3). Sows from CAT3 had the highest CL number and final sow score (*p* < 0.05) and presented the worst values for total piglets born, piglets born alive, low-weight piglets born ( < 1 kg), proportion of low-weight piglets born per litter, and mean litter weight at birth. However, stress biomarker levels did not differ among categories and were not associated with litter performance.

**Conclusions:**

Our findings indicate that severe CLs impair litter performance and increase litter heterogenicity, particularly the incidence of low-weight piglets born. These effects do not appear to be directly linked to stress biomarker levels but may result from behavioral and physiological disruptions affecting animal well-being derived from the presence of severe CLs. Given the significant economic and welfare implications of severe CLs, further research is needed to elucidate their impact on reproduction and to develop effective protocols to better detect stress and pain in affected sows.

## Background

Claw lesions (CLs) are multifactorial pathologies characterized by structural alterations (erosion, rupture, or overgrowth) in the wall, heel, sole, heel-sole junction, or white-line of the pig’s foot [1, 2]. The reported prevalence of these lesions in sow herds ranges between 50 and 100%, with most sows displaying at least one lesion that vary in type and severity [3–5]. Claw lesions are among the most relevant factors affecting both the welfare and profitability of breeding sows [5]. Mild CLs may be asymptomatic, whereas moderate to severe lesions contribute to 5–24% of lameness cases, the second most common cause of early culling [1, 3, 6–8]. Early culling increases production costs per weaned piglet and reduces overall herd productivity [3, 5, 7, 9]. Even in the absence of overt lameness, CLs may cause pain and discomfort [10, 11], leading to reduced activity and standing time, decreased feed and water intake, and compromised nutritional status. This, in turn, may impair reproductive performance [6, 9, 12]. Claw lesions have been associated with key reproductive indicators, including increased wean-to-first service intervals, reduced numbers of live-born and weaned piglets, lower weaning weights, and higher risks of stillbirth and piglet mortality due to crushing [10, 13]. These effects are particularly pronounced in sows with severe lesions on the heel, sole, wall, or white line [7, 9, 11].

Persistent CLs may also induce chronic stress, as observed in cows [14]. Chronic stress can impair reproductive performance by redirecting energy towards vital processes and altering gonadotropin secretion through increased cortisol levels [15, 16]. Maternal stress during pregnancy can also negatively affect offspring health and behavior, reducing resilience and compromising its future development [15, 17, 18]. Studies in humans and ruminants indicate that maternal stress is a major risk factor for low birth weight [19, 20]. This is particularly relevant to pig production, given the current high incidence of heterogeneous litters with a substantial proportion of low-weight piglets born (LWPB) [21]. Despite its significant impact on pre-weaning mortality and production efficiency [22], LWPB is often overlooked when assessing the effects of CLs in pregnant sows. Thus, investigating whether CLs in pregnant sows are associated with an increased incidence of LWBP may provide a deeper understanding of their consequences for litter quality and overall productivity.

European Union legislation mandates group housing for sows from four weeks after artificial insemination (AI) until one week before farrowing [23]. While this practice has welfare benefits, it also increases the prevalence of CLs due to aggression and competition during feeding [4, 12]. Given the high prevalence of CLs in breeding sows and their direct and indirect effects on welfare, reproductive performance, and productivity, effective claw health management is essential to maintain herd profitability.

The primary objective of this study was to evaluate the impact of CLs on prolificay of pregnant sows, with a particular focus on litter heterogeneity. Since we hypothesized that CLs induce chronic stress, which negatively affects reproductive performance and increases litter heterogeneity, particularly the incidence of LWPB, three specifc objectives has been proposed: (1) analyze the prevalence and characterize the types and severity of CLs in pregnant sows, (2) determine the association between CLs and stress biomarkers, and (3) evaluate the effects of CLs on litter heterogeneity, focusing on LWPB incidence.

## Methods

The study was conducted over a 10-month period on a commercial pig farm located in the southeastern Spain (Agropor SL, Murcia, Spain), which operates under a continuous improvement plan for animal welfare and holds the Interporc Animal Welfare Spain (IAWS) certificate and the Welfare Quality (IRTA) certificate, based on the European Welfare Quality and AWIN® standards.

### Animals and housing

Sows included in this study were weaned hyper-prolific sows (TN70, Topigs Norsvin©), with a mean parity number of 3.0 ± 0.13 (range 1 to 7). All sows had comparable lactation periods (22.0 ± 0.04 days; range: 21 to 24) and a mean body condition of 2.9 ± 0.01 (range 2.7 to 3.2) on a five-point scale at weaning, where 1 corresponds to emaciated, 2 to thin, 2.5 to thin but still acceptable, 3 to ideal, and 4–5 to fat to obese; scores between 2.5 and 3.0 are considered adequate score for weaned sows. Semen for AI was obtained from sexually mature Duroc boars from AIM Iberica, an insemination center in Murcia, Spain. Individual AI crates measured 60 cm in width, 244 cm in length, and 109 cm in height. Each crate had a solid concrete floor in the front section and slatted concrete flooring in the rear. Gestation pens provided a total area of 2.2–2.5 m^2^ per sow and featured a combination of solid and slatted concrete flooring, with at least 60% of the surface being solid. Individual farrowing crates measured 2.4 m × 1.8 m. In this case, floor consisted in slatted flooring under the sow and solid concrete flooring in the piglets area. Shredded newspaper was used as bedding material for piglets being changed twice a day. Environmental conditions in the AI pens and farrowing crates were controlled to ensure consistent temperature and humidity levels. Sows had ad libitum access to water and were fed once daily, at early morning, with a diet formulated to meet their nutritional requirements.

### Reproductive management

Estrus detection began one day after weaning and was performed daily by a skilled operator using a vasectomized boar to stimulate behavioral signs. Females exhibiting a standing reflex were considered to be in estrus, with this day designated as Day 0. All sows were inseminated at 6 and 24 h after the onset of estrus using a post-cervical AI procedure (40 mL of AI dose containing 1.5–2.0 × 10^9^ spermatozoa). Signs of estrus were observed daily from Day 18 to Day 24 after AI. At 24 days post-AI, pregnancy was confirmed via transabdominal ultrasonography (MyLab X1, Esaote, Genova, Italy).

### Claw lesions examination

**Fig. 1:**
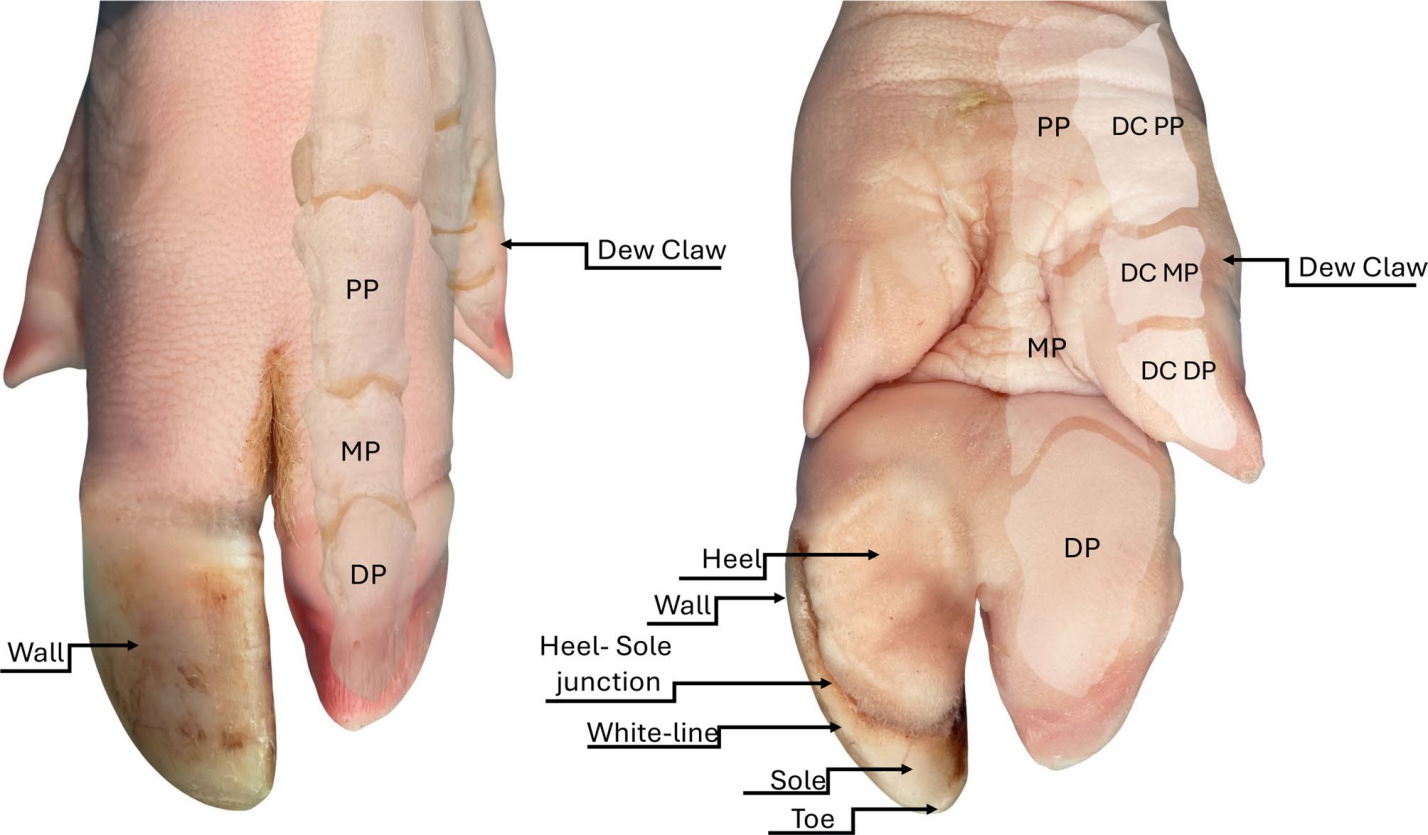
Frontal and rear pig foot views, showing the anatomical areas evaluated for each claw. Wall: the hard outer layer of each claw, can also be called the hoof horn. Heel: soft keratinized epidermis on the volar surface of the claw. Sole: the bottom portion of the claw, it is slightly softer than the wall and in pigs covers a relatively small area. Heel-sole junction: juncture line between the heel and the sole. White line: the line around the edge of the sole that is the junction between the sole and the wall on the underside of the claw. The toe: the anterior part of the sole. (PP, MP and DP: proximal, medial and distal phalange of the main digit; DC PP, DC MP and DC DP: proximal, medial and distal phalange of the dew claw) [1, 24, 25]

Two trained observers examined the front and hind claws of each sow. The anatomical areas of the claw were defined according to Anil et al. [1] and included the wall (hard outer layer), heel (soft keratinized epidermis on the ventral surface), sole (hard keratinized area anterior to the heel), heel-sole junction, white line (junction between the sole and wall) and toe (anterior part of the sole) (Fig. 1). Claw lesions were classified following the Feet First protocol from Zinpro Corporation [26] according to affected area: abnormal toe length (ATL), overgrowth of dew claw (ODC), heel overgrowth and erosion (HOE), fissure of the heel-sole junction (FHSJ), white line injuries (WLI), and horizontal or vertical cracks on the wall of the hoof (HCW and VCW). Location of the CLs in the lateral or medial claw for all four feet was also recorded.

### Hair sampling and cortisol, cortisone and oxytocin extraction

Hair samples were collected from the croup region between 9:00 and 10:30 a.m, following the protocol described by Lopez-Arjona et al. [27, 28], with minor modifications. Briefly, hair was shaved close to the skin in both sides (right and left) of the croup region, avoiding dirty or contaminated areas. Hair samples were stored in hermetically sealed plastic bags and refrigerated until analysis. Cortisol, cortisone, and oxytocin extraction followed the method described by Lopez-Arjona et al. [27, 28]. Briefly, 250 mg of hair was washed twice with isopropanol (5 mL) and centrifuged at 1,500 × g for 1 min. The pellets were washed again with isopropanol, under the same conditions, and dried at room temperature. Hair samples were cut with scissors into small pieces (approximately 0.5 cm) and 60 mg of each sample were pulverized until a fine powder was obtained, using a homogenizer (Precellys Evolution homogenizer, Bertin Technologies, Montigny-le-Bretonneux, France). Subsequently, steroid and oxytocin extraction was performed in pulverized samples by incubation with 1 mL methanol during 18 h at room temperature under continuous gentle agitation. Samples were then centrifuged (200 × g, 5 min) and a volume of 0.6 mL of each methanol extract was aliquoted into a new Eppendorf tube. These aliquots were evaporated to dryness using a Speed Vac Concentrator (Concentrator 5301, Eppendorf, Hamburg, Germany) and the dry extracts will be reconstituted with 0.1 mL of PBS and stored at −80 °C until analysis.

### Cortisol, cortisone and oxytocin analysis

Cortisol and cortisone concentrations were measured using AlphaLISA^®^ competitive assays (PerkinElmer Inc., Waltham, MA, USA) validated for porcine hair [27]. Cortisol analysis was performed using a mouse monoclonal antibody (MA1–16703, Thermo Scientific), while cortisone assays used a custom rabbit polyclonal antibody. Oxytocin was measured using a direct competitive AlphaLISA^®^ assay with a monoclonal antibody obtained previously by Lopez-Arjona et al. [29]. All assays were performed in 96-well plates with a total volume of 50 µL per well. The intra- and inter-assay coefficients of variation for cortisol, cortisone, and oxytocin were 6.3%, 7.0%, and 3.2%, and 6.7%, 8.5%, and 9.1%, respectively, with limits of quantification of 7.0, 2.8, and 0.2 pg/mg of hair, respectively.

### Litter performance

The prolificacy parameters assessed were total piglets born, piglets born alive, stillborn piglets, and mummified piglets. Immediately after parturition, each piglet was individually weighed.

### Experimental design

**Fig. 2:**
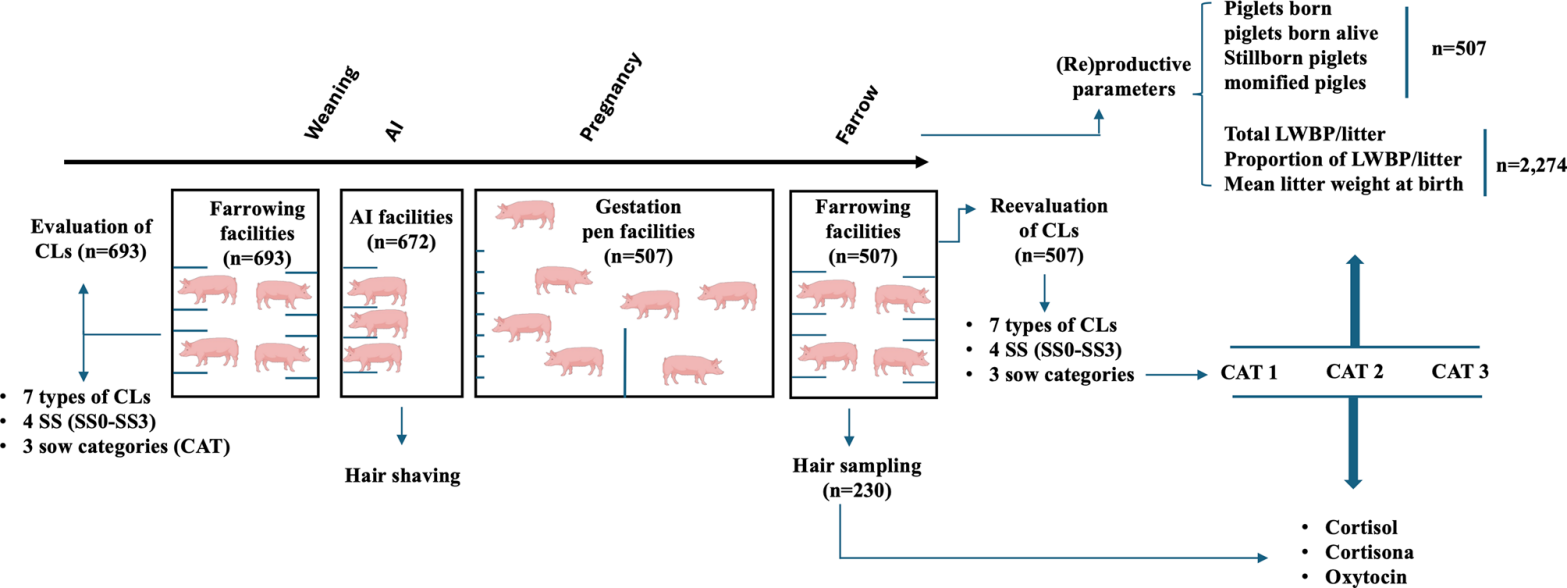
Experimental design. The study involved 693 hyper-prolific sows, initially evaluated for claw lesions. After weaning, sows were housed in individual AI crates, where their hair was shaved to ensure that hair samples collected at the end of the subsequent gestation period reflected newly grown hair. These samples were subsequently used for biomarker assays. Twenty-eight days post-insemination, pregnant sows were transferred to group gestation pens, and one week prior to the expected farrowing date, they were moved to individual farrowing crates. Claw lesions were reevaluated in the farrowing crates after farrowing, and each sow’s claws were scored for no lesions (SS0) to severe (SS3) lesions. Sows were classified into three categories based on lesion severity: CAT1, CAT2, and CAT3. Prolificacy parameters, along with piglet birth weight (normal or low-weight), were recorded. The number of low-weight piglets born (LWPB) per litter, their proportion, and mean litter weight were calculated for each sow category

A total of 693 hyper-prolific sows were initially enrolled in the study and assessed for CLs at farrowing. Following weaning, all sows were housed in individual AI crates, and their hair was shaved to ensure that the hair samples collected at the end of the subsequent gestation period corresponded to newly grown hair. However, sows that did not exhibit adequate body condition at weaning or failed to conceive after AI were excluded from further analysis. Consequently, 507 pregnant sows were ultimately included in the study. At 28 days post-insemination, these sows were moved to group gestation pens (10–15 sows per group), and approximately one week before the expected farrowing date (based on the AI date), they were transferred to individual farrowing crates following routine pre-farrowing cleaning procedures. At this point, newly grown hair samples from randomly selected sows (*n* = 230) were shaved and collected for biomarker analysis. Claw lesions were reassessed post-farrowing to prevent any disturbance to the sows during the birthing period, ensuring that sows remained calm and lying down during the evaluation. When necessary, front and hind claws were washed prior to clinical evaluation. Each CL was scored for severity (SS) on a scale of 0 to 3: SS0, no lesions; SS1, mild lesions (defined as slight, short, superficial or very small superficial defects or deviations from a healthy claw); SS2, moderate lesions (defined as longer or larger but shallow lesions), and SS3, severe lesions (more serious and deeper lesions, including severe ulceration extending into the chorion). Since all sows with lesions SS2 also showed SS1 lesions and all sows with lesions SS3 also displayed SS1 and SS2 lesions, three sows categories were established as follows: CAT1 consisted of sows with no lesions or at least one SS1 lesions; CAT2 included sows with SS1 and at least one SS2 lesions, but not SS3 lesions; and CAT3, comprised sows with SS1, SS2 and at least one SS3 lesions. A final sow score (FSS) was calculated by summing the points assigned to all lesions observed in an individual sow. Lesions were assigned 1, 2, or 3 points according to their severity, corresponding to SS1, SS2, and SS3 lesions, respectively. For example, a sow with four SS1 lesions would have an FSS of 4. In contrast, a sow with four SS1 lesions, two SS2 lesions, and one SS3 lesion would have an FSS of 11. After farrowing, prolificacy parameters were recorded. A total of 2,274 piglets were classified according to birth weight as either normal piglets (NP: weight ≥1 kg) or LWPB ( < 1 kg), given that piglets below this threshold, particularly in hyperprolific sow lines, have been shown to be at increased risk of preweaning mortality [30]. The average number of LWPB per litter, proportion of LWPB per litter and mean litter weight at birth were calculated for each sow category (CAT1, 2, and 3). The experimental design is summarized in Fig. 2.

### Statistical analysis

Data were analyzed statistically using the SPSS program version 28 (IBM, Chicago, IL, USA). The normality of the variables was determined using the Kolmogorov-Smirnov test. For normally distributed data (litter weight at birth) comparisons among groups were performed using a mixed model (GLMM), which included the fixed effect of CL category (three levels: CAT1, CAT2, and CAT3) and the random effect of the batch. When ANOVA revealed significant effects, post-hoc comparisons were conducted using the Bonferroni test. The Kruskal-Wallis test was applied to non-normally distributed data (parity, lactation length, body condition, pregnancy length, piglets born, piglets born alive, stillborn piglets, mummified piglets, LWPB, proportion of LWPB, and cortisol, cortisone and oxytocin levels). Significance values were adjusted using the Bonferroni correction for multiple comparisons. The Chi-square test, with Yates’ correction when appropriate, was used to compare percentage data. The results are expressed as percentages or as means ± standard error (SEM). Differences were considered significant when the p-value was below 0.05.

## Results

No significant differences were observed among sow categories in parity number (3.2 ± 0.1, 2.9 ± 0.2 and 3.0 ± 0.1), lactation length (22.3 ± 0.07, 22.3 ± 0.06 and 21.9 ± 0.06 days), body condition score (2.9 ± 0.01, 3.0 ± 0.01 and 3.1 ± 0.01) or pregnancy length (115.2 ± 0.2, 115.1 ± 0.1 and 115.4 ± 0.2). These values correspond to categories 1, 2, and 3, respectively.

**Fig. 3:**
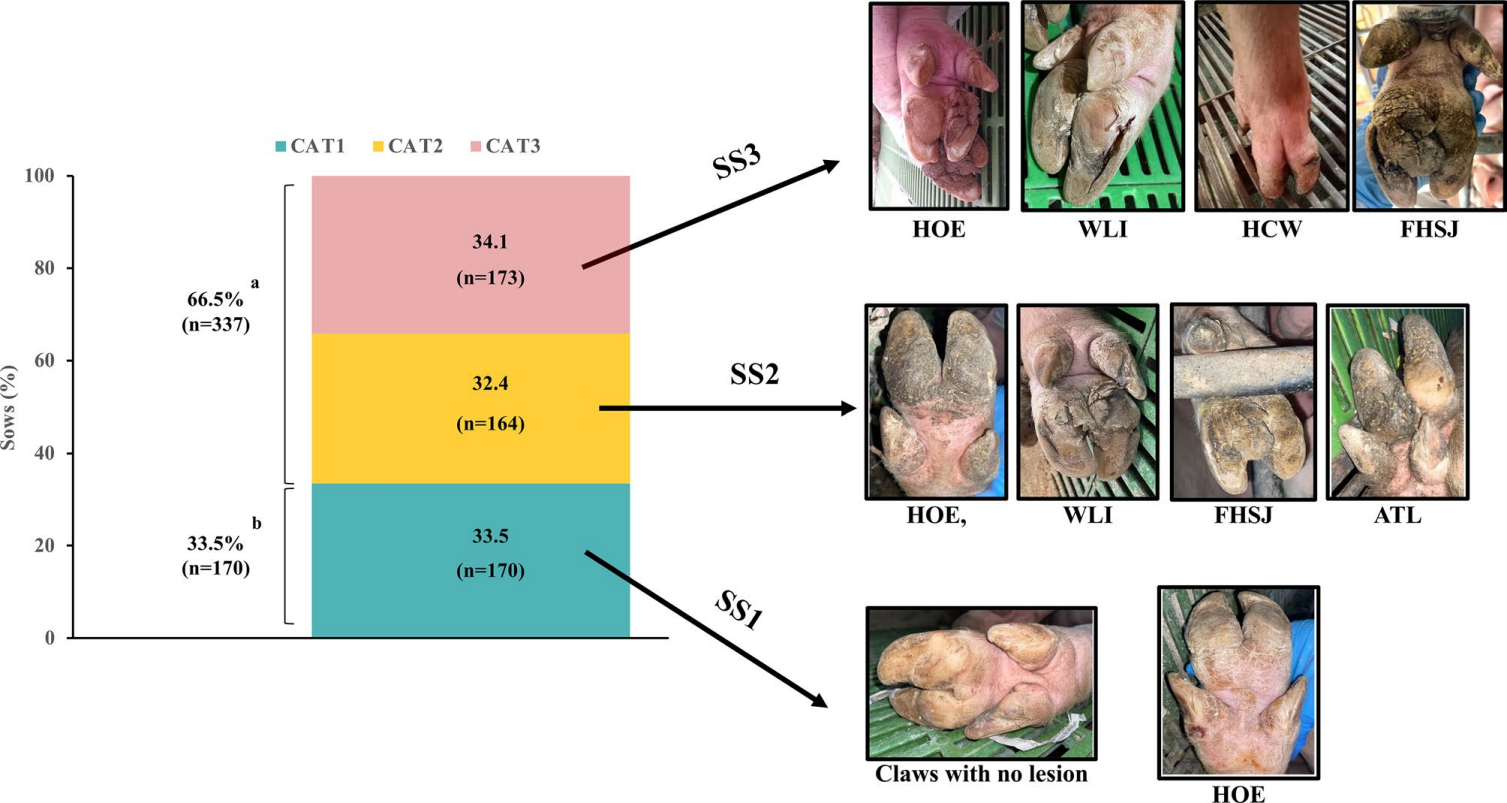
Distribution of sow categories with representative images illustrating claw lesion types and severity scores. Claw lesion types: HOE, heel overgrowth and erosion; WLI, white line injuries; HCW, horizontal cracks on the wall; FHSJ, fissure of the heel-sole junction; ATL, abnormal toe length. Severity scores (SS) were defined as follows: SS0, no lesions; SS1, mild lesions (slight, short, superficial or very small superficial defects or deviations from a healthy claw); SS2, moderate lesions longer or larger but shallow lesions); and SS3, severe lesions (serious and deeper, including severe ulceration extending into the corium). Sows were classified into 3 categories (CAT): CAT1 (SS0+SS1), CAT2(SS1+SS2, not SS3), and CAT3 (SS1+SS2+SS3). ^a,b^ *p* < 0.001

The results indicated that only 4.1% of the evaluated sows were free from CL. These unaffected sows, along with those exhibiting mild lesions, were classified as CAT1, comprising 33.5% of the total sow population (Fig. 3). The remaining sows were nearly equally distributed between CAT2 (32.4%) and CAT3 (34.1%). These percentages were similar between categories. Together, 66.5% of the sows displayed more serious lesions (moderate and severe; CAT2 + CAT3), a percentage significantly higher (*p* < 0.001) than the 33.5% of sows showing no or only mild lesions (CAT1).

**Fig. 4:**
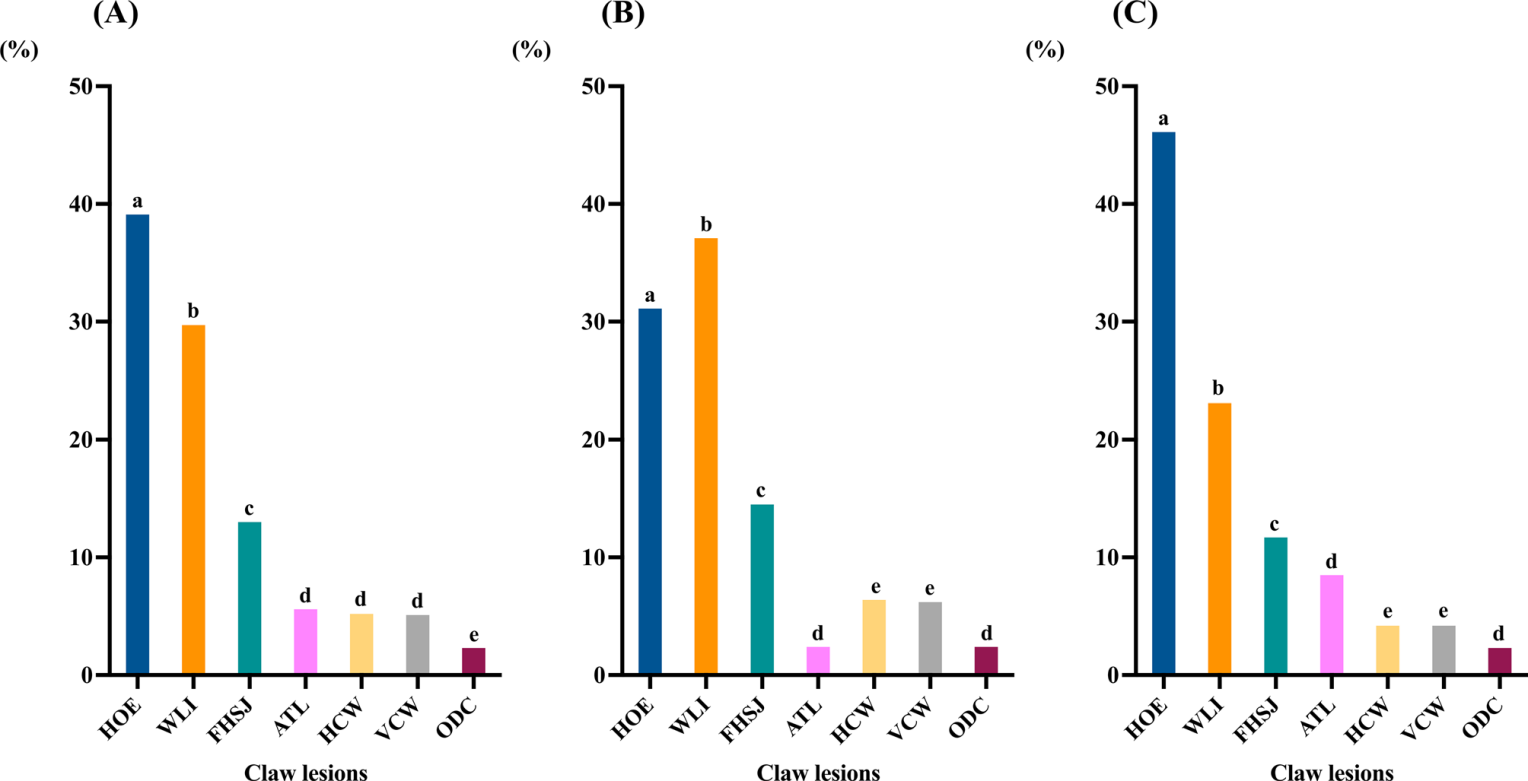
Frequency distribution of different types of claw lesions evaluated. (**A**) frequency of claw lessions (CLs) relative to the total number of observed lesions (*n* = 2,900). (**B**) frequency of CLs in the forelimbs (calculated from the total number of lesions observed in the forelimbs only; *n* = 1,362). (**C**) frequency of CLs in the hindlimbs (calculated from the total number of lesions observed in the hindlimbs only; *n* = 1,538). (HOE) heel overgrowth and erosion; (WLI) white line injuries; (FHSJ) fissure of the heel-sole junction; (ATL) abnormal toe length; (HCW) horizontal cracks on the wall; (VCW) vertical cracks on the wall; (ODC) overgrowth of the dew claw. Different letters (a, b, c, d, e) in the same graphic indicate significant differences (*p* < 0.01)

The proportional distribution of CL types, irrespective of sow category and anatomical location (forelimbs or hindlimbs), is presented in Fig. 4A. Among all recorded CLs (*n* = 2,900), the most frequently observed lesion (39.1%, *p* < 0.001) was HOE, followed by WLI (29.7%), and FHSJ (13.0%). Dew claw overgrowth, which accounted for 2.3% of the total observed CLs, was the least prevalent lesion. The prevalence of CLs was higher (*p* < 0.001) in the hindlimbs (53.1%) than in the forelimbs (46,9%). In both locations, lateral claws were more affected than medial claws (93.1% vs 6.9% in hindlimbs compared to 73.7% vs 26.2% in forelimbs, *p* < 0.01). Heel overgrowth and WLI were the most frequently observed lesions in both forelimbs and hindlimbs (*p* < 0.001), albeit with differing distributions. In the forelimbs, WLI were the most frequent lesion, where as in the hindlimbs, heel overgrowth was predominant. Fissures at the heel-sole junction were the third most frequent CL in both locations. Furthermore, significant differences (*p* < 0.001) were observed in the distribution of less frequent lesions between forelimbs and hindlimbs (Fig. 4B and C).

**Fig. 5:**
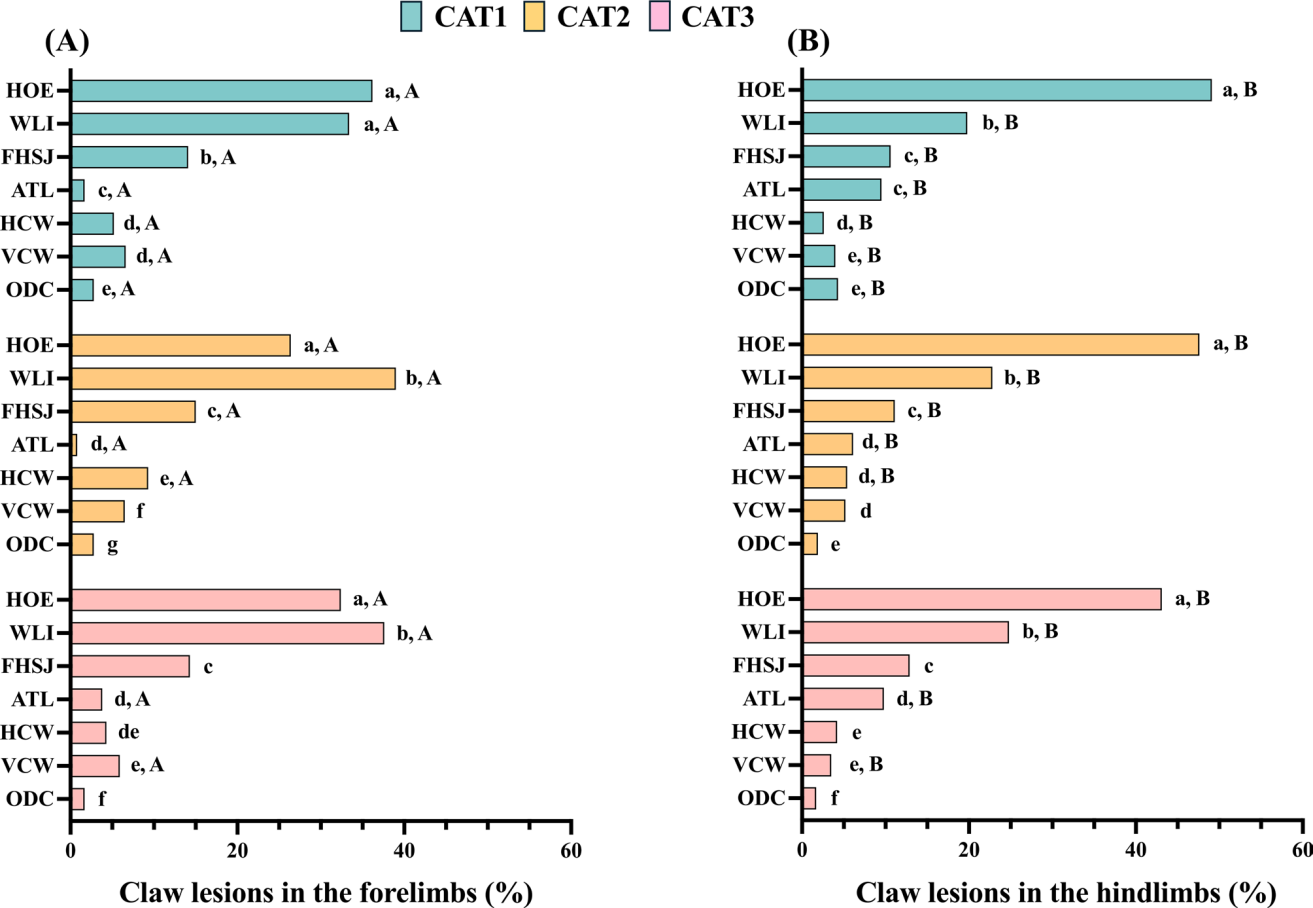
Distribution of claw lesion (CL) types by sow category (CAT1, CAT2, CAT3) and anatomical location. (**A**) frequency of each CL type relative to the total CL recorded in the forelimbs (*n* = 1,362) by sow category. (**B**) frequency of each CL type relative to the total CL recorded in the hindlimbs (*n* = 1,538) by sow category. a,b,c,d,e,f indicate significant differences (*p* < 0.05) among CL types within each category; A,B indicate significant differences among CL types within each category between forelimbs and hindlimbs

Among the total CLs evaluated, 22.0% were observed in sows classified as CAT1, whereas higher proportions (*p* < 0.001) were observed in CAT2 (35.6%) and CAT3 (42.4%). This pattern remained consistent when CLs were analyzed separately in the forelimbs (21.3%, 36.1% and 42.6% for CAT1, CAT2, and CAT3, respectively) and hindlimbs (22.6%, 35.1% and 42.3% for CAT1, CAT2, and CAT3, respectively). In the forelimbs, HOE and WLI were the most prevalent lesions (*p* < 0.001) irrespective of sow category. The proportions of these lesions were comparable between CAT2 and CAT3, where the prevalence of WLI was significantly higher (*p* < 0.001) than that of HOE. Conversely, in CAT1, the prevalence of HOE and WLI was similar. In the hindlimbs, HOE was the most frequently observed CL across all three categories (*p* < 0.001). Significant differences were observed in the percentages of CLs between forelimbs and hindlimbs for most CL types (Fig. 5A and B).

**Fig. 6:**
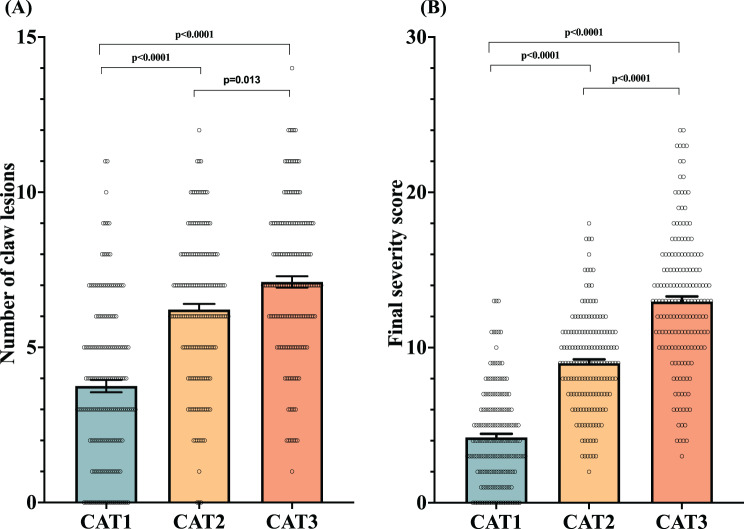
Number of claw lesions and final severity score per sow according to sow category (CAT). (**A**) the number of claw lesions (CLs) per sow was determined by summing the CLs observed in the claws of both forelimbs and hindlimbs, regardless of severity score. (**B**) the final severity score was calculated by summing the severity scores of all CLs present in the claws of the forelimbs and hindlimbs for each sow

The mean number of CL per sow and the FSS were analyzed for each sow category (Fig. 6 A and B, respectively). Significant differences (*p* < 0.05) were observed among the three categories for both parameters. Sows in CAT1 consistently exhibited the lowest values (3.7 ± 0.2 and 4.2 ± 0.2 for mean CL count and FSS, respectively), whereas those in CAT3 showed the highest values for both CL count (7.1 ± 0.2) and FFS (12.9 ± 0.3).

Analysis of hair cortisol, cortisone, and oxytocin levels revealed no significant differences among the three sow categories (Table 1).

**Table 1:** Mean values ± SEM of stress biomarkers in sow hair samples (*n* = 230) by category

Parameter (pg/mg)	Sow category		
	CAT1 (*n* = 70)	CAT2 (*n* = 83)	CAT3 (*n* = 77)
Cortisol	55.6 ± 4.0	36.8 ± 6.4	49.7 ± 3.1
Cortisone	142.2 ± 11.6	122.2 ± 20.1	152.62 ± 10.2
Oxytocin	3.9 ± 0.2	3.0 ± 0.5	4.1 ± 0.3

CAT1: sows with no lesions or at least one mild lesion; CAT2: sows with mild lesions and at least one moderate lesions, but not severe lesions; CAT3: sows with mild lesions, moderate lesions and at least one severe lesion.

Data from the corresponding farrowing revealed that sow category significantly influenced (*p* < 0.05) several key parameters. Specifically, total piglets born, piglets born alive, the number and proportion of low-weight piglets per litter, and average litter weight were all affected. Sows classified as CAT1 showed improved values across all these parameters compared to those in CAT3, while CAT2 exhibited intermediate values that did not differ significantly from either CAT1 or CAT3, except for the proportion of low weight piglets (Table 2). No other significant differences were found for the remaining recorded parameters.

**Table 2:** Reproductive performance of sows classified by categories according to the severity of claw lesions

Parameter	Sow category		
	CAT1 (*n* = 170)	CAT2 (*n* = 164)	CAT3 (*n* = 173)
Piglets Born	14.1 ± 0.3^a^	13.9 ± 0.3^ab^	12.8 ± 0.3^b^
Piglets Born Alive	13.1 ± 0.3 ^a^	13.0 ± 0.3 ^ab^	11.9 ± 0.3 ^b^
Stillborn Piglets	0.9 ± 0.1	0.7 ± 0.1	0.8 ± 0.1
Mummified Piglets	0.15 ± 0.05	0.23 ± 0.05	0.17 ± 0.04
Low Weight Piglets Born*	0.8 ± 0.2^a^	2.2 ± 0.3^ab^	2.3 ± 0.4^b^
Proportion of Low Weight Piglets*	0.05 ± 0.01^a^	0.15 ± 0.02^b^	0.15 ± 0.02^b^
Mean Litter Weight at birth*	1.5 ± 0.04^a^	1.40 ± 0.03^ab^	1.38 ± 0.03^b^

CAT1: sows with no lesions or at least one mild lesion; CAT2: sows with mild lesions and at least one moderate lesions, but not severe lesions; CAT3: sows with mild lesions, moderate lesions and at least one severe lesion. * Data collected from 167 farrowings, comprising a total of 2,274 piglets that were individually weighed (CAT1: 49 sows/687 piglets; CAT2: 65 sows/894 piglets and CAT3: 53 sows/693 piglets). ^a,b^ in the same row indicate significant differences (*p* < 0.05). Values are means ± SEM.

## Discussion

The results of this study reveal a high prevalence of CLs in breeding sows and provide evidence of a significant association between severe CLs and the impairment of key litter performance parameters. Furthermore, this research is the first to suggest a potential link between severe CLs in pregnant sows and increased litter heterogeneity, specifically a higher proportion of LWPB. The findings suggest that presence of severe CLs may alter the physiological status of pregnant sows and could represent an important factor contributing to the high incidence of LWPB, which remains a major concern in modern swine production systems.

The results of the present study indicated that most sows (95.9%) had at least one CL regardless of severity. Moderate to severe CLs were observed in 66.5% of sows, a proportion that falls within the lower range of previous studies, which have reported prevalence rates as high as 100% [4, 5]. A key factor influencing our results is the sow housing system. Following European Union regulations established in 2013 [23], sows in the present study were housed in small, stable groups from 28 days post-insemination until one week before farrowing, in order to minimize inter-animal aggression [15]. Although this system may reduce skin and CLs compared to dynamic groups (large number of animals and continuous entry of new animals), the etiology of CLs is multifactorial, involving genetic, nutrition, management, housing, disease, age and body weight [5, 31]. Therefore, the consistency of our findings with prior research highlights the widespread occurrence of CLs and the need for further investigation into contributing factors.

Prevalence analysis showed that volar surface lesions (HOE, FHSJ and WLI) were the most common. Claw lesions were slightly more prevalent in hindlimbs than forelimbs, with lateral claws significantly more affected than medial claws. The findings are consistent with previous studies and can be explained by well-known factors such as weight distribution, flooring, housing dynamic, claw biomechanics and environmental factors [1, 3, 11, 13, 32]. Among these factors, weight distribution is critical, where 75% of a sow’s weight being borne by the lateral digits, which increase the risk of lesions, especially on lateral claws [1, 33], as seen in the current study. The combination of unbalanced weight loading, hard flooring and softness of specific claw zones further heighten susceptibility to injury [13]. It should be highlighted that besides its higher prevalence, CLs are linked to lameness, a major welfare and economic concern for swine producers [34, 35]. Therefore, the implementation of strategies to reduce CL incidence is essential for improving sow welfare and productivity.

Of all lesions evaluated, the highest proportion (42%) was observed in sows classified as of CAT3. CAT3 sows also exhibited a higher number of lesions per individual and a greater FSS than CAT1 and CAT2 sows. These results suggest that severe foot lesions may increase the risk of additional CLs. Previous research indicate that CL-association pain and inflammation can significantly alter a sow postural behavior [36], resulting in increased weight-bearing on the contralateral limb and potentially the formation of new foot lesions. In this study, severe lesions were more prevalent than mild or moderate lesions, contrasting with Fitzgerald et al. [11] and Kramer et al. [3], who reported higher mild or moderate lesion rates. This discrepancy likely reflects the multifactorial nature of CLs [3]. Interestingly, despite the high frequency of severe lesions, only 3.7% of the sows evaluated exhibited lameness (data non show). This agrees with previous reports of high CL prevalence without concurrent lameness [2, 10, 37]. However, these lesions may still cause pain and discomfort, potentially compromising welfare and health.

Our findings indicate that severe CLs negatively impact relevant farrowing parameters. Sows with severe lesions produced approximately one fewer piglet in total and live births, compared to those with no or mild lesions. Similar associations have been reported previously [9, 38]. Recent studies also link severe WLI and HOE to poorer farrowing performance in Brazilian commercial herds [39]. In contrast, we did not find association between severe CLs and stillborn piglets, differing from previous reports [7, 39]. Differences in study design, CL evaluation methods, farm-specific factors, and sow characteristics likely contribute to discrepancies across studies.

Despite this variability across studies, it is evident that severe CLs negatively impact reproductive parameters, highlighting the need to elucidate the underlying mechanisms. One potential explanation involves chronic stress resulting from prolonged pain. Einarsson and colleagues [40] reported that chronic stress significantly affects reproductive indexes by influencing the hypothalamic-pituitary-adrenal axis. To explore this hypothesis, we measured stress biomarkers in sow hair. While previous research suggests that hair cortisol concentrations may be useful for assessing stress in pigs [15], the literature presents contradictory findings regarding its reliability. In the present study, hair cortisol levels in CAT3 sows were similar to those in CAT1 and CAT2 sows. This observation supports two potential interpretations: first, that hair cortisol may not serve as a reliable biomarker for assessing stress under the conditions of this study, consistent with previous reports indicating its limited utility as a stress indicator in certain sceneraies [15, 41]; or second, that CAT3 sows were not subjected to significant stress. Hair cortisone and oxytocin, both identified in previous studies as potential stress biomarkers [27, 28], were included in the study due to their proposed roles in modulating the physiological stress response; however, their levels showed no significant variation between sow categories. Consequently, no direct link could be established between severe CLs and stress-induced reproductive alterations.

An alternative mechanism underlying the impairment of litter performance in sows from CAT3 may involve inflammation induced by severe lesions. Severe CL are deep and can penetrate the corium, triggering an inflammatory response associated with pain, anorexia, lethargy, and altered behavior (e.g. reduced standing time). Such changes can decrease water and feed intake, ultimately impairing reproductive function [6, 9, 11, 39].

Interestingly, beyond the impact of severe CLs on farrowing parameters, our results revealed differences in litter performance, particularly in the incidence of LWPB. Concretely, sows with severe CLs have a high number and proportion of LWPB per litter compared to those with no or mild lesions, resulting in lower mean litter birth weight. The presence of LWPB is a determining factor in the farm productivity, particularly in intensive pig production systems [42]. Recent literature indicates that the presence of these piglets may be attributable to the use of hyperprolific sows, as these sows produce larger, but more heterogeneous, litters, resulting in greater presence of LWPB [43]. In our study, all sows evaluated were hyperprolific, genetically similar, and had comparable parity. Therefore, the increased LWPB in CAT3 sows is likely influenced by other factors. Given that CL severity and number were the only distinguishing factors between CAT1 and CAT3 sows, an association between severe CLs and LWPB incidence is plausible. Therefore, chronic stress may be discarded as a primary cause, suggesting that discomfort from severe CLs could be the main driver. As previously discussed, severe CLs may lead to reduced standing and feed intake, impairing maternal nutrition and delaying fetal growth, thereby increasing litter heterogenicity and LWPB [44]. While previous studies have linked severe foot lesions to reduced litter weight at weaning [11], this is the first study to suggest a potential association between severe CLs and increased litter heterogeneity due to LWPB.

A recent study reports that moderate lameness in pregnant sows impairs placental function, reducing its ability to protect fetuses from maternal stress, which implies that fetuses are exposed to elevated cortisol levels, a catabolic agent that restricts fetal growth and tissue development [45]. Given the high incidence of CLs in breeding farms, the increased stress sensitivity of hyperprolific sows [46], and evidence from humans and ruminants studies linking maternal stress to impaired placental function and low birth weight [19, 20] further research on that topic is mandatory. Development of protocols for accurately detecting stress and pain in sows with severe CLs, along with assessing placental function, could significantly enhance understanding of the mechanisms by which these lesions impair (re)productive performance.

## Conclusions

Our findings confirm the high prevalence of CLs in breeding sows, with severe lesions on the volar surface of lateral hind claws being the most common. Notably, severe CLs were directly associated with impaired litter performance, including fewer total and live-born piglets. Additionally, severe CLs appeared to increase litter heterogeneity through the presence of LWPB, likely due to alterations in sow behavior and physiological status. While the reduction in piglet numbers is a direct effect of severe CLs on pig farm productivity, the increased risk of lameness and higher incidence of LWPB have indirect consequences. Lameness compromises sow longevity and productivity, often leading to premature culling, while LWPB face developmental challenges higher pre-weaning mortality and suboptimal growth. Given the substantial economic and welfare implications of severe CLs, further research is crucial to develop effective preventive strategies and management practices, ensuring both animal welfare and the sustainability of swine production systems.

## Data Availability

No datasets were generated or analysed during the current study.
